# Supplementary material for: Effect of opioid-free anesthesia combined with pectoral nerve block on the quality of recovery in patients after mastectomy: A randomized, controlled trial
Source: PLoS One. 2025 Apr 3;20(4):e0320592. doi: 10.1371/journal.pone.0320592 (PMC11967923; doi:10.1371/journal.pone.0320592)
Supplement: Attachment — Submitted filename: Response to Reviewers.docx [file pone.0320592.s004.docx]

Rebuttal Letter to PLOS ONE

PLOS ONE Editorial Office

PLOS ONE

Dear Dr. De Cassai and the PLOS ONE Editorial Team,

Thank you for the opportunity to revise our manuscript titled "Effect of opioid-free anesthesia combined with pectoral nerve block on the quality of recovery in patients after mastectomy: A randomized, controlled trial" (Manuscript ID: PONE-D-24-44917). We appreciate the constructive feedback provided by you and the reviewers, which has helped us improve the quality of our work.

Below, we address each of the reviewers' comments and outline the changes made to the manuscript:

# Reviewer #1 Comments:

We appreciate the reviewer's positive feedback regarding the statistical presentation and study design. We have ensured that all statistical analyses are clearly described in the revised manuscript.

# Reviewer #2 Comments:

## 1. “*Firstly, the main outcome of the paper is not so innovative since ERAS protocols already promotes opioid alternatives to provide opioid reduced anesthesia (ORA) and OFA to patients.*”

- We acknowledge the concern regarding the innovation of our study. While ERAS protocols do promote opioid alternatives, our research specifically investigates the effects of opioid-free anesthesia (OFA) combined with regional blocks on the quality of recovery (QoR) after mastectomy. This focus on OFA in breast surgery is novel and provides specific data on its impact on postoperative recovery, pain management, and adverse events. Importantly, our study has already implemented several core measures of ERAS, including multimodal analgesia, PONV prevention, and total intravenous anesthesia (TIVA), which is recommanded by ERAS sociaty[1]. These elements not only enhance the QoR but also increase the relevance of our study as a reference for the implementation of ERAS protocols in clinical practice.

## 2. “*Secondly, the reason why your main outcome refers to a study that doesn’t include mammary surgery is not clear (Bu eat al. “Validation of the Chinese version of the Quality of Recovery-15 Score and Its Comparison with the Post-Operative Quality Recovery Scale.”). Furthermore, in my opinion, the value of QoR-15 you used as baseline (106,5) is very low and does not represent the real quality of recovery of patients undergoing mastectomy beyond the use of opioids. This is also clear from your results: the lowest value of QoR-15 you have registered is 127.*”

- We selected Bu et al.'s study[2] as a reference because it validated the Chinese version of the QoR-15 and was relevant to our patient population in mainland China. We clarify that Bu's study does include breast surgery, categorizing it under “General” surgery type in Table 1 in their study. Our findings of a higher postoperative QoR-15 score (127.4±14.0) compared to Bu's baseline score (106.5) reflect advancements in perioperative management, particularly in minimizing opioid use. We have recalculated the required sample size based on the QoR-15 scores from Zhang et al.'s study[3], which reported a postoperative 24-hour QoR-15 score of 132.0 ± 12.0 for patients receiving opioid-based anesthesia (OBA). Our sample size has been recalculated based on this new reference, ensuring that our study is adequately powered to detect clinically meaningful differences.

# Sample Size Calculation:

Using the formula for comparing two means in clinical trials:

$n_{A}=\kappa n_{B}$ and $n_{B}=\left( 1+\frac{1}{\kappa} \right){(\sigma\frac{z_{1-\alpha/2}+z_{1-\beta}}{\mu_{A}-\mu_{B}})}^{2}$

$1-\beta=\Phi(z-z_{1-\alpha/2})+\Phi(-z-z_{1-\alpha/2})$, $z=\frac{\mu_{A}-\mu_{B}}{\sigma\sqrt{\frac{1}{n_{A}}+\frac{1}{n_{B}}}}$.

with a MICD of 8, SD of 12, and a significance level of 0.05. Therefore, we propose a total sample size of 74 patients (37 per group) to detect a clinically meaningful difference in QoR-15 scores. This revised calculation ensures that our study is adequately powered to assess the impact of OFA combined with regional blocks on the quality of recovery in patients undergoing mastectomy.

## 3. “*In the end, I think it would’ve been interesting to analyse the variation between the QoR-15 before the surgery and after to evaluate if there were any differences between the two groups.*”

- We have added an analysis of the variation in QoR-15 scores before and after surgery in Table 3. The results show no statistically significant difference between the two groups, with changes of -13.4±10.1 in the OFA group versus -12.2±12.2 in the OBA group (MD=-1.2, P=0.54).

## 4. **Minor Revisions**:

We have made the following revisions as per your suggestions:

‘*At page 5, lines 19-20; you should specify the type of surgery maybe writing “undergoing total or partial mastectomy, with axillary lymph node dissection or sentinel lymph node biopsy”;*’

- Specified the types of surgeries performed (total or partial mastectomy, with axillary lymph node dissection or sentinel lymph node biopsy) on Page 5, Line 19-20.

“*At page 6, lines 20-21; even if implicit, you should explain better the type of block you performed and the dosage and type of anaesthetic you used for each injection;*”

- Provided a detailed explanation of the PECS block technique, including the dosage and type of anesthetic used, on Page 6, Line 20-22.

“*At page 7, talking about muscle relaxation, include the neuromuscular monitoring;*”

- Included a statement regarding the lack of neuromuscular monitoring on Page 7, Line 16-18.

‘*At page 8, clarify what you did include in “adverse events”;*’

- Clarified the definition of "adverse events" on Page 9, Line 1-2.

“*In table n.1 you should also consider the QoR-15 pre-surgery;*”

- Updated Table 1 to include pre-surgery QoR-15 scores.

“*Consider a rearrangement of the Discussion paragraph as it’s too long compared to the overall length of the article.*”

- Rearranged the Discussion section for improved clarity and conciseness on Page 18-20.

We believe these revisions adequately address the reviewers' concerns and enhance the quality of our manuscript. We appreciate the opportunity to improve our work and look forward to your feedback on our revised submission.

Thank you for your consideration.

Sincerely,

Qingyan Luo

From the Department of Anesthesiology, Obstetrics and Gynecology Hospital of Fudan University, Shanghai, China.

Reference

1.	Temple-Oberle C, Shea-Budgell M, Tan M, Semple J, Schrag C, Barreto M, et al. Consensus Review of Optimal Perioperative Care in Breast Reconstruction: Enhanced Recovery after Surgery (ERAS) Society Recommendations. Plastic and reconstructive surgery. 2017;139(5):1056e-71e.

2.	Bu X, Zhang J, Zuo Y. Validation of the Chinese Version of the Quality of Recovery-15 Score and Its Comparison with the Post-Operative Quality Recovery Scale. The patient. 2016;9(3):251-9.

3.	Qingfen Z, Yaqing W, Haiyan A, Yi F. Postoperative recovery after breast cancer surgery: A randomised controlled trial of opioid-based versus opioid-free anaesthesia with thoracic paravertebral block. Eur J Anaesthesiol. 2023;40(8).
